# Variations in the Behavior of Pigs During an Open Field and Novel Object Test

**DOI:** 10.3389/fvets.2020.00607

**Published:** 2020-09-03

**Authors:** Amy Haigh, Jen-Yun Chou, Keelin O'Driscoll

**Affiliations:** ^1^Pig Development Department, Animal and Grassland Research and Innovation Centre, Teagasc, Moorepark, Fermoy, Ireland; ^2^Royal (Dick) School of Veterinary Studies, University of Edinburgh, Easter Bush, United Kingdom; ^3^Animal and Veterinary Sciences Research Group, Roslin Institute Building, Scotland's Rural College (SRUC), Easter Bush, United Kingdom

**Keywords:** tail biting, fear, reactive, proactive, cortisol

## Abstract

Tail biting is a serious welfare concern in pig production. It not only causes distress for victims, but may occur where pigs are unable to cope, and become biters. An animal's ability to cope with stressful situations may vary between individuals, but the behavioral response could be consistent across different fear eliciting situations. We exposed 75 pigs to open field (OF) and novel object (NO) tests at 14 weeks of age. Within each pen of pigs (*n* = 16 pens, 55 pigs/pen), 6 pigs were selected for testing using the following criteria: 3 pigs that had severe bite wounds (BITTEN), 1 confirmed biter (BITER), 1 pig which could be easily approached and trained to provide a saliva sample (BOLD) and 1 pig which was extremely evasive, and was unable to be trained to willingly provide a saliva sample (SHY). Given that responses may be consistent in different scenarios, we hypothesized that SHY pigs would display more characteristics of a fear response (i.e., less movement in the open field, more time spent by the door, and longer latency to approach the novel object) than human BOLD pigs. We also hypothesized that BITTEN pigs would behave similarly to SHY and BITERS similarly to BOLD. The BOLD and BITER pigs spent more time exploring (*P* < 0.05) and less time by the door (*P* < 0.01) than the BITTEN and SHY pigs. Although there was an overall increase in cortisol level from before to after the tests (*P* < 0.001), this was only significant for BITTEN (*P* < 0.001) and SHY (*P* < 0.05) pigs. Therefore, as hypothesized, for several measures, BOLD, and BITER pigs behaved similarly, and differently to SHY and BITTEN. However, the low sample size potentially meant that for several measures, although numeric differences were in the direction hypothesized, there were no statistical differences. Further work in which a greater number of BITER pigs were included in the sample, may elucidate our hypotheses more clearly, as to whether responses to fear tests in pigs could be associated with the likelihood of being a tail biter, or victim.

## Introduction

Tail biting is one of the most significant problems in pig production, as it causes pain and distress for victims (1) and financial loss for producers (2). The problem has a multifactorial etiology, with damaging biting behavior thought to occur through three main mechanisms: “two-stage,” which consists of mild chewing escalating to severe biting (thought to arise from a lack of manipulable material), “sudden-forceful,” which is associated with competition for access to resources, and “obsessive” whereby the causal factors are not known, but the biter moves from pig to pig, compulsively biting tails (3).

Open Field (OF) and Novel Object (NO) tests are often used to study fearfulness in pigs, and indeed have previously been used to compare the responses of tail biters, tail bite victims, and non-affected animals (4–6). In general, these tests have shown that biters and/or victims react differently to “control” pigs both behaviourally (biter pigs less likely to approach a NO (4) more time lying and walking, less standing still, lower latency to touch the object (6), and physiologically (e.g., dysfunctional autonomic regulation (6), in ways which indicate that the animal is fearful. However, it is unclear as to whether these differences relate to a generalized fearfulness, or whether they are specific to exposure to this type of test. Moreover, in these studies, the control pigs were pigs which were not affected by biting, rather than having been selected because they have a propensity to react in a fearful or confident manner to novel or fear eliciting situations.

Pigs can respond differently depending on the type of behavioral test which is applied; this is because tests of fearfulness can comprise of varying levels of fear eliciting stimuli, such as novelty, physical proximity, movement, intensity, suddenness, and duration (7). Pig level characteristics such as their age and sex can also influence the response (8), as well as their personality, which is generally considered to consist of individual differences which are consistent over time and situations (9). Thus, pigs which respond fearfully or with confidence during any particular situation, may not display the same characteristics in another.

This experiment was carried out as a component of a larger study comparing compressed straw blocks or plastic toys as enrichment for pigs from weaning to finish (10). The aim of the work was to initially investigate whether pigs which were human BOLD in their home pen (could be approached/would willingly approach people, and could easily be trained for cortisol collection) would respond in a less fearful manner to both an open field (OF) and novel object (NO) test, than pigs which were human SHY in the home pen (couldn't be approached/wouldn't willingly approach people and couldn't easily be trained for cortisol collection but would consistently run away) (i.e., was their behavior consistent across the two scenarios). We then investigated whether pigs which were either tail BITERs or BITTEN would respond differently to the tests. Given that tail biting is associated with increased stress levels in victims (11) we hypothesized that BITTEN pigs would display behavior similar to SHY pigs (i.e., a reduced ability to respond to a fear eliciting situation), whereas BITER pigs would respond similarly to BOLD, as the novel situation would provide an outlet for exploratory behavior (12).

## Methods

The study was conducted on a commercial farm, ~10 miles from the Animal and Grassland Research and Innovation Center, Teagasc Moorepark Fermoy, Co. Cork, Ireland. Prior to commencing the experiment approval was obtained by Teagasc Animal Ethics Committee (TAEC89/2015).

### Animals and Treatments

A total of 880 pigs (Terminal line PIC, born from Large White X Landrace sows) which were born in two replicates 7 weeks apart (440 per replicate) were used in the experiment. Approximately 75% of the tail was docked at 3 days of age (as per veterinary recommendation at the farm), and males were kept intact. Piglets were weighed and individually ear tagged just prior to weaning and split into 16 groups of 55 piglets on the basis of sex and weight. Piglets of the same sex and from the same litters were kept together, to minimize stress due to re-mixing at weaning. Only piglets in excess of 5 kg and devoid of any injury were included in the experiment. Piglets were weaned at 28 days of age, assigned to treatment, and were managed in the same group until the end of the 2nd stage weaner accommodation.

Details of the experiment are published in the paper comparing compressed straw blocks and plastic toys with regard to damaging behavior, by Haigh et al. (10). In brief, the experiment followed a 2 × 2 factorial design with sex (male or female; *n* = 8 pens each) and enrichment (compressed straw (STRAW) or plastic hanging toys (TOY); *n* = 8 pens each) as the main factors. Both types of enrichment (STRAW or TOY) were provided at an allowance of two devices per pen (i.e., ~27 pigs per device). The straw blocks were cylindrical (~3 inches diameter), and provided in a dispenser (a plastic cylinder (Medinova, Italy) with an open bottom into which the straw block fitted snugly) attached to the wall of the pen. The straw block was supported by a metal bar suspended under the cylinder, so that ~4 inches of straw were exposed to the pigs. These cylinders were 30.6 inches in length, and were placed 8 inches from the floor in the 1st stage and 19 inches in the 2nd stage weaner pens, and straw was provided continuously.

In the TOY treatment, pigs were provided with a different type of toy in the 1st stage and 2nd stage weaner pens, appropriate to the age of the pig. In the 1st stage the toy consisted of a Porchichew (Ketchum, U.K) suspended at two points in the center area of the pen. In the 2nd stage weaner pens pigs were provided with a rubber hanging “Easyfix” chew toy (Easyfix rubber products, Canada), and a hanging plastic barrel, again both suspended in the center area of the pens.

### Selection of Pigs for Behavior Tests

Six pigs per pen were selected to take part in the open field (OF) and novel object (NO) tests; 1 pig which were consistently “human bold” (BOLD), 1 pig which were consistently “human-shy” (SHY), 1 pigs which was confirmed as an active tail biter (BITER), and 3 pigs which had severe lesions on either the ear or tail (BITTEN). Pigs were only used that corresponded to one of the categories i.e., no bitten/biter pigs had also been trained for cortisol collection.

### Bold and Shy

Pigs were identified as being BOLD or SHY using data collected as part of the larger study (10). For that study saliva was collected from a subsample of 11 focal pigs from each pen (176 pigs in total) every fortnight from 2 weeks post weaning, to investigate salivary cortisol level. These pigs were selected on the basis of weaning weight; pigs were sorted by weight and every 5th pig selected, so that the full range of weights in the pen were represented. During the first week post-weaning these 11 pigs per pen were habituated to saliva collection by gradually introducing the cotton buds (Salivette, Sarstedt, Wexford, Ireland) used for collection and the experimenter; pigs were to chew on the cotton bud until it was thoroughly moistened (about 30–60 s/sample). As well as the habituation period, four saliva collection days occurred prior to OF and NO testing. If a pig displayed fearful behavior (i.e., ran away, and/or refused to chew on the cotton bud) collection did not occur that week. Pigs that displayed this behavior at least three times prior to behavioral testing were categorized as SHY, and pigs that habituated extremely quickly and gave a good sample voluntarily each time were categorized as BOLD. Within each pen one SHY and one BOLD pig was selected for testing.

### Biter Pigs

During the larger study (10), direct behavior observations were conducted each week. The selection of biter pigs for the OF and NO test were therefore based on a total of eight of these observations, 5 during the first weaner stage (9 ± 2, 14 ± 0, 20 ± 2, 28 ± 1, and 38 ± 4 days post weaning) and 3 during the second weaner stage (46 ± 4, 51 ± 5, and 59 ± 4 days post weaning. Observations were carried out four times on each day between 11:30 (hh:mm) and 15:30. Three trained observers collected all the behavior data, balanced across enrichment type and pens. Observation times were also balanced across enrichment type and pens so that observations for each pen were distributed approximately equally across the recording periods. The behavior of the entire group was observed for 5 min. All incidences of harmful and aggressive behavior and play were counted using continuous observation, with tail and ear biting being defined as oral manipulation of the tail or ear of another pig (3). During these observations, the identity of individuals engaged in ear and tail biting was recorded. Over a total of 85 h of observation (~5 h per pen), a total of 500 records of individuals biting were collected. Within each pen, a BITER was selected that was observed biting other pigs' tails or ears on at least three occasions (Mean-3.8 ± 1.64 (SD), range = 3–9), and more than any other pig in the pen, during these observation sessions. Priority was given to pigs observed biting both ears and tails and observed biting in both the first and second stage indicating it being a consistent trait and not an occasional occurrence, Sniffing around the tail or ear area was not considered biting, the pig had to have the tail or ear in the mouth. Biting was further authenticated by a jolt or flinch in the victim of the biting.

### Bitten Pigs

The tails and ears of all pigs were examined individually by the experimenter walking through the pen, on a fortnightly basis, including the week immediately before behavioral testing. This would have been conducted five times prior to the OF and NO, three in the first weaner stage weaner (18 ± 0, 31 ± 4, and 43 ± 4 days post weaning) and two in the second weaner stage (55 ± 6 and 73 ± 9 days post weaning). Tail lesions were scored using the tail lesion scoring system developed by Hunter et al. (13) (0 = no damage, 1 = mild, 2 = moderate, and 3 = severe lesions). Ear lesions were scored using a modified version of the system developed by Haigh et al. (10), and range from 0 (perfect) to 4 (part of the ear missing). Pigs that had a score of 3 or more to either the tail or at least one ear were categorized as bitten pigs. As per standard farm management, all pigs considered unwell or lame were removed to hospital pens and therefore removed from the experiment.

### Open Field (OF) and Novel Object (NO) Tests

Testing took place over five consecutive days per replicate when pigs were in 2nd stage weaner pens, ~70 days after weaning. Test pigs were removed from their home pen, and a saliva sample collected immediately in the corridor outside the pen. The pig was then moved to a waiting area, along with at least one companion pig, immediately adjacent to the test arena. As soon as the preceding pig was finished its test and removed, the pig entered the arena alone and testing began. A pig was never held in the waiting area for more than the entire duration of testing of the preceding pig. Thus, individual pigs were kept outside their home pen for a maximum of 10 min, including collecting saliva collection, immediately prior to the test.

The test arena was an unused room on the commercial farm, which was a maximum of 10 m from the pigs' home pens, with a solid concrete floor and concrete walls, measuring ~3 × 3 m. The door into the room consisted of a gate 130 cm in height, which was covered by a wooden panel prior to testing. This was so that when the gate was closed the pigs could not see out of the room. The observer stood outside the room and slightly to the left of the gate, which allowed her to see over the top of the gate and have a full view of the room, without entering it. One corner of the room had a series of metal posts installed in the floor diagonally across the corner, so these were also covered with a wooden panel (thus the room was not entirely square). When testing was complete the observer entered the room and collected a second saliva sample, prior to the pig being brought back to its home pen.

The floor of the test arena was divided into nine areas so that the amount of movement through the room during the tests could be assessed. Behavior (Table 1) was recorded by a single observer using the Psion Workabout with observer software (Noldus Information Technology, Wageningen, The Netherlands). The OF test began as soon as the pig entered the test arena. After the pig had spent 5 min in the arena, a novel object was presented to the pig in the form of a red yard brush with a rope attached that was lowered over the gate. The pig was then observed for a further 3 min in the presence of this novel object, using the same ethogram as before but with additional behaviors directed toward the object being also recorded. The duration of the behavioral tests was therefore a maximum of 8 min.

**Table 1:** Ethogram of behaviors recorded by continuous observation during the open field and novel object tests.

**Behavior type**	**Behavior**	**Description**
State	Stand	Stationary with all four feet on the floor
	Lie	Stationary with body in contact with the floor
	Walk	Moving in a forward or backward direction or turning around at the same location, with head up
	Exploration	Investigating the floor, wall, or object with the rooting disc. See detailed descriptions below
	Explore floor	Investigating the floor by sniffing, nosing, licking, rubbing, or rooting it with the rooting disc. Pig is oriented toward the center of the room. Rooting disc is either in contact or very close to floor surface.
	Explore wall	Investigating the walls of the arena by sniffing, nosing, licking, rubbing, or rooting it with the rooting disc. Rooting disc is either in contact or very close to wall surface
	Self-groom	Scratching or stimulating a part of the body using the fixtures or fittings
	Play	Individual play behavior, including scampering, jumping/running around
	Attention object^*^	Attention is directed toward object but the pig has not yet touched it
	Withdraw^*^	Drawing back from object with or without touching it, while attention is still directed toward it
	Explore object^*^	Investigating the object by sniffing, nosing, licking, rubbing, carrying, throwing, or rooting it with the rooting disc. Rooting disc is either in contact or very close to the object
Event	Low-pitched vocalization	Short or long grunts
	High-pitched vocalization	Screams, squeals, or grunt-squeals
	Elimination	Defecating or urinating
	Jump	Jumping in air or against a wall of the arena

* *indicates behaviors which were only recorded during the novel object test*.

The ethogram of behaviors recorded for the OF and NO tests is shown in Table 1. For the OF test, behaviors considered for analysis were the amount of time spent walking, performing exploratory behavior (wall and floor), standing still, and the number of vocalizations (low pitched and high pitched). Additionally transitions between the different areas of the room were counted to provide an estimate of the amount of movement around the pen. The percentage time spent in the squares immediately next to the door was also investigated. The number of pigs which eliminated and attempted to escape were also counted.

For the NO test the main focus of interest was on interaction with the novel object. Thus, the behaviors considered for analysis were the time spent directing attention toward the object, interacting with the object, withdrawing from the object, and the latency to interact with it.

### Cortisol Analysis

Immediately after cortisol collection the cotton buds were placed in plastic tubes and refrigerated, then centrifuged for 15 min at 3,000 g, and stored at −20°C until analyzed by an enzyme linked immunosorbent assay (Salivary Cortisol Kit, Salimetrics Europe Ltd, Suffolk, U.K). The minimum detectable concentration of cortisol that could be distinguished from 0 was <0.003 mg/dl. The intra- and inter-assay CV's based on controls were 7.2 and 12.9%, respectively.

### Data Management

Due to varying levels of tail biting in the pens, in the second replicate it was not possible to select the planned number of pigs within each type from each pen, as the categories were not all mutually exclusive (e.g., BOLD pigs were also BITTEN etc.). These pigs were removed from the analysis. The final number of pigs which were available for analysis within each type are shown in Table 2 (Total, *n* = 75 pigs).

**Table 2:** Numbers of pigs selected within each type for open field and novel object testing.

**Type**	**Description**	**Rep 1**	**Rep 2**	**Total**
Bold	Pigs quickly habituated quickly to saliva collection, and saliva was successfully collected on four collection days (as per 10)	8	4	12
Shy	Saliva not successfully collected in any, or less than half of collection days. The pig consistently ran away when approached during these sessions	8	4	12
Biter	Pig observed biting other pigs' tails or ears on at least three occasions (range 3–9), and more than any other pig in the pen, during weekly observation sessions over a 5 week period (as per 10)	8	4	12
Bitten	Either the tail or ear badly bitten (bleeding with open wound) a maximum of 1 week prior to testing	24	15	39

### Statistical Analysis

Data were analyzed using the Statistical Analyses System (SAS, V9.1.3, SAS Institute Inc., 1989). Data were investigated for skew, kurtosis, and outliers before analysis by examination of box and normal distribution plots.

Behavior data were initially explored using correlations (Pearsons and Spearmans Rank, where appropriate) and principle component analysis (PCA; Proc Factor), with the aim of determining whether some of the recorded behaviors clustered and share the same underlying motivation. For the PCA, the numbers of animals which eliminated and jumped were not included due to the an extremely high number of animals eliminating (*n* = 69), and low number jumping (*n* = 4). A varimax rotation was used, as components were considered to be orthogonal. Components were considered meaningful by considering whether the eigenvalue was >1, evaluation of the Scree plot, the proportion of variance explained by components individually and combined, and by considering the interpretation of the output (14).

Data for the OF and NO tests were subsequently analyzed separately using linear mixed models, where residuals confirmed this was appropriate. The model included fixed effects of pig type (*n* = 4; BOLD, SHY, BITER, or BITTEN), treatment (*n* = 2; STRAW or TOY), sex (*n* = 2; male or female), and replicate (*n* = 2; 1 or 2). Pen and the day on which the test was carried out were included as random effects. To investigate the hypotheses that BOLD and BITER behaved differently to SHY and BITTEN pigs, a contrast statement was used. For this comparison, we hypothesized that the BOLD and BITER pigs would behave as a single cohort (labeled BRAVE) and the SHY and BITTEN pigs would behave differently to BRAVE, but similarly to each other, so were also considered a single cohort (labeled SCARED). Where data were not appropriate for analysis using linear models, the Kruskall-Wallis test was used instead (number of screams, attention toward the novel object, and time spent withdrawing from the object). The number of pigs performing these behaviors, or not, were also compared using Fishers exact test.

For analysis of salivary cortisol, a similar linear mixed model to that described above was used, with the addition of sampling time (before or after the behavior tests) as a repeated measure. The random effect of plate was also included in this analysis.

In all analysis using linear models, residuals were examined to verify normality and homogeneity of variances. Differences in least squares means were investigated using the *t*-test, using Tukeys adjustment for multiple comparisons. Degrees of freedom were estimated using Kenwood–Rogers adjustment. Differences were considered significant at *P* ≤ 0.05. Tendencies toward significance (0.05 < *P* ≤ 0.10) are also presented. Data are presented as Least Squares means ± SE.

## Results

### Correlations

All correlations and their significance levels are shown in Table 3. In brief, exploration (explore floor, wall, or object in Table 1) was negatively correlated with walking, standing still, the number of locations entered, the duration spent at the door, and the number of screams. Grunts and screams were positively correlated. The number of locations traversed was positively correlated with grunts and screams, yet negatively correlated with the number of eliminations. The duration by the door was positively correlated with both grunts and screams. The number of grunts was negatively correlated with the latency to interact with the object, and positively correlated with the time spent interacting with it. The time directing attention toward the object was positively correlated with the latency to interact with it, and the time spent withdrawing from it, and negatively correlated with the time spent interacting with it. The time spent interacting with it was conversely negatively correlated with the time spent withdrawing and the latency to interact.

**Table 3:** Correlations between the behaviors measured in the open field and novel object tests.

	**Walking^a^**	**Exploration^a^**	**Standing^a^**	**No locations^a^**	**Duration by door^a^**	**Grunts^a^**	**Screams^b^**	**Elimination^a^**	**Attention^b^**	**Interaction^a^**	**Withdraw^b^**	**Latency to interact^b^**
Walking^a^	**1**	**−0.668 <0.001**	0.003 0.98	**0.638** **<0.001**	0.190 0.10	**0.283** **=0.01**	**0.353** **=0.001**	−0.175 0.13	−0.016 0.88	−0.044 0.71	−0.007 0.95	–**0.194** **<0.05**
Exploration of floor, wall or object^a^		**1**	–**0.598** **<0.001**	–**0.227** **=0.05**	–**0.460** **<0.001**	−0.194 0.10	–**0.347** **<0.01**	0.162 0.17	0.118 0.32	0.101 0.39	0.103 0.38	0.075 0.41
Standing^a^			**1**	**−0.371** **<0.001**	0.065 0.58	**–**0.155 0.18	**–**0.082 0.48	0.015 0.90	0.049 0.68	**−0.270** **<0.05**	**–**0.025 0.83	**0.209** **<0.05**
No. locations^a^				**1**	0.113 0.34	**0.278** **<0.05**	**0.229** **<0.05**	**−0.225** **=0.05**	**–**0.047 0.69	0.008 0.95	−0.008 0.95	**-0.219** **0.06**
Duration by door^a^					**1**	**0.233** **0.042**	**0.375** **<0.001**	**–**0.112 0.338	0.012 0.92	0.128 0.27	**–**0.156 0.18	**–**0.085 0.47
Grunts^a^						**1**	**0.340** **<0.01**	0.054 0.65	**–**0.142 0.23	0.128 0.28	**–**0.185 0.11	**−0.272** **<0.05**
Screams^b^							**1**	**–**0.062 0.60	**–**0.038 0.74	0.031 0.79	**–**0.131 0.27	**–**0.171 0.15
Elimination^a^								**1**	0.151 0.20	0.068 0.57	**–**0.051 0.67	**–**0.126 0.29
Attention^b^									**1**	**−0.235** **<0.05**	**0.276** **<0.05**	**0.328** **<0.01**
Interaction^a^										**1**	**−0.283** **<0.05**	**−0.596** **<0.001**
Withdraw^b^											**1**	0.103 0.378
Latency to Interact^a^												**1**

a *Correlations carried out using Pearsons correlation coefficient (r)*.

b *Correlations carried out using Spearmans rank correlation coefficient (r_s_)*.

*Significance levels are indicated below correlation coefficients, and significant relationships highlighted in bold*.

### Principle Component Analysis

When the 11 behavior variables were included in an initial PCA, the first three components had an eigenvalue >1 and the scree plot indicated that there was a large separation between component three and four. These initial three components contributed 24.3, 18.2, and 15.0% of the variance in the dataset (combined: 57.4%). However, the variable “exploration” was found to load onto both component 1 and 3, and as such the analysis was re-run after removal of this variable. After the new analysis, the first three components still had an eigenvalue >1, there was a clear separation between component 2 and 3 according to the scree plot. These three components contributed 23.8, 18.6, and 12.5% of the variance in the dataset, totalling 54.5%. However, “walk” the number of locations visited, and the time spent withdrawing from the object now loaded onto two components, and as such were removed from a third analysis. This analysis showed that 3 components with an eigenvalue >1, yet no clear separation between any components. Components 1, 2, and 3 contributed 27.8, 20.1, and 14.7% respectively, totalling 62.6%), but only two variables loaded onto components 2 and 3. Moreover, the three remaining variables in the dataset which were contributed from the novel object test (attention toward the object, latency to touch the object, and time spent interacting with it) all loaded on to component 1. As such, the variables which contributed to the PCA did not meaningfully reduce the dataset into a smaller number of components which could be compared.

### Open Field Test

**Figure 1:**
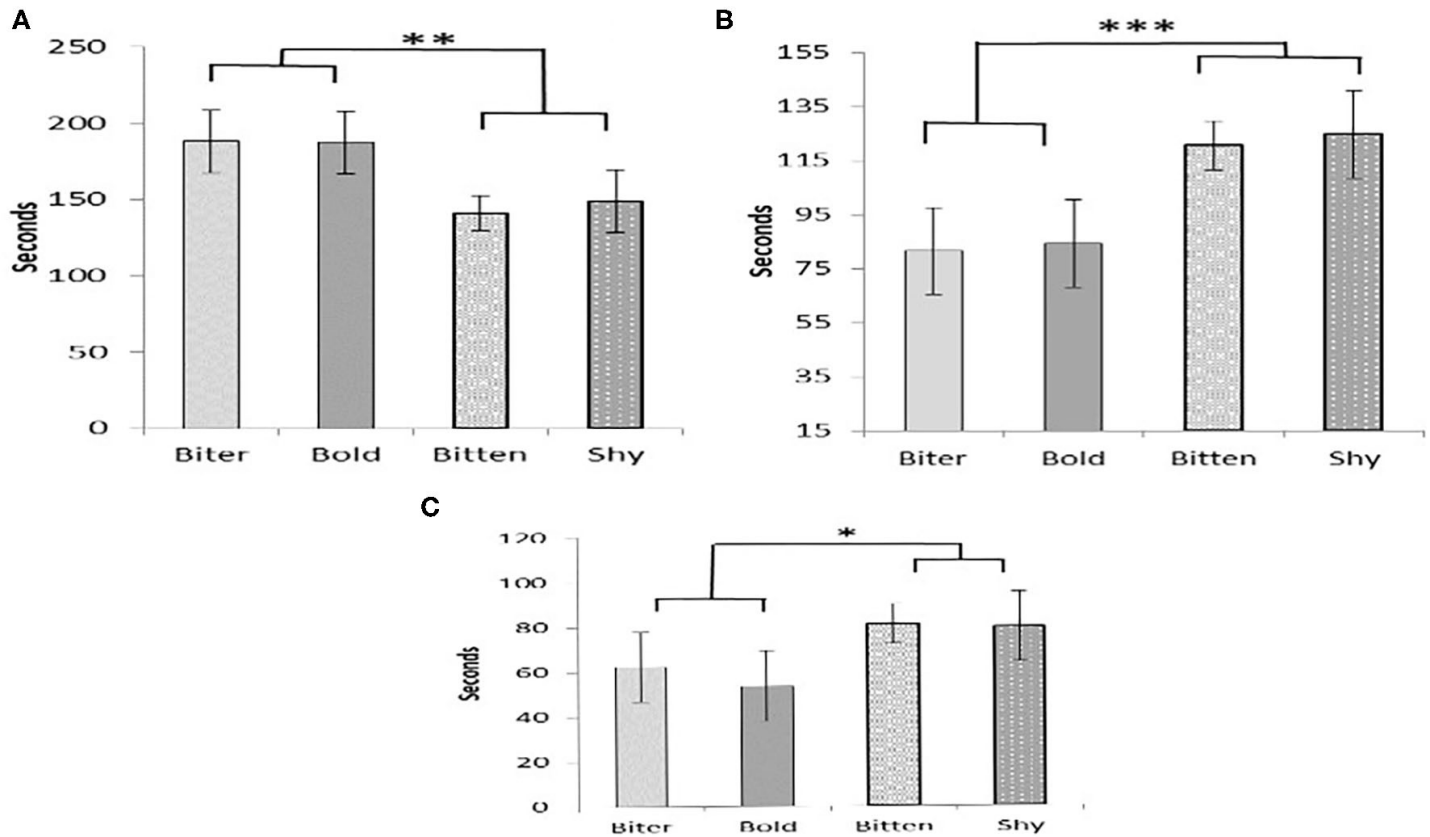
The amount of time pigs in each category spent exploring **(A)**, by the door **(B)**, and standing still **(C)**. Tendencies and significant differences between BITER and BOLD with BITTEN and SHY pigs are indicated by * = 0.5 < *P* ≤ 0.1; ** = 0.01 < *P* ≤ 0.05, *** = 0.001 < *P* ≤ 0.01.

There were no pair-wise differences between the pig type categories in any of the measurements taken during the OF test (Table 4). However, there tended to be an overall effect of pig type on the time spent exploring (*P* = 0.09) and there was also an effect on the time spent by the door (*P* = 0.05). For both of these, there was a difference between the pigs that were SHY or BITTEN pigs when compared to those that were BOLD or BITER pigs (Figures 1A,B). The BOLD and BITER pigs spent more time exploring (*P* < 0.05) and less time by the door (*P* < 0.01) than the SHY and BITTEN pigs (Figures 1A,B). The SHY and BITTEN pigs also tended to spend more time standing still than the BITER and BOLD (*P* = 0.1; Figure 1C).

**Table 4:** Results from the open field test and novel object test.

	**BOLD**	**SHY**	**BITER**	**BITTEN**	***P*-value**
**Open field test**					
Walk	00:33 ± 00:11	00:39 ± 00:11	00:35 ± 00:11	00:42 ± 00:06	0.87
No. square transitions	23.12 ± 3.06	23.95 ± 3.06	23.95 ± 3.06	24.91 ± 1.71	0.96
Low grunts	38.22 ± 5.85	79.55 ± 5.85	33.63 ± 5.85	33.30 ± 3.37	0.63
Screams^a^	17% [0 (0–0)]	8% [0 (0–0)]	% [0 (0–0)]	23% [0 (0–0)]	0.38
Elimination	3.24 ± 0.47	1.74 ± 0.47	1.66 ± 0.47	2.40 ± 0.26	0.06
**Novel object test**					
Attention^a^	58% [2.8 (0.26–7.8)]	75% [1.34 (0–6.0)]	67% [2.2 (0–6.1)]	56% [2.1 (0–9.4)]	0.87
Latency to interact	00:51 ± 00:19	01:11 ± 00:19	01:12 ± 00:19	00:48 ± 00:10	0.55
Interaction duration	01:46 ± 00:17	00:57 ± 00:17	01:12 ± 00:17	01:11 ± 00:09	0.19
Withdraw^a^	33% [0 (0–8.7)]	42% [0 (0–1.76)]	17% [0 (0–0)]	10% [0 (0–0)]	0.10

*Unless indicated otherwise, data are presented as Least Squares means ± standard error*.

a *Data presented as the percentage of pigs which performed the behavior, as well as the median and interquartile range of the duration for which it was performed, including 0 values. P-values are in relation to the Kruskall-Wallis test*.

### Novel Object Test

There was no effect of pig type on any of the measurements recorded during the NO test (Table 4).

### Salivary Cortisol

**Figure 2:**
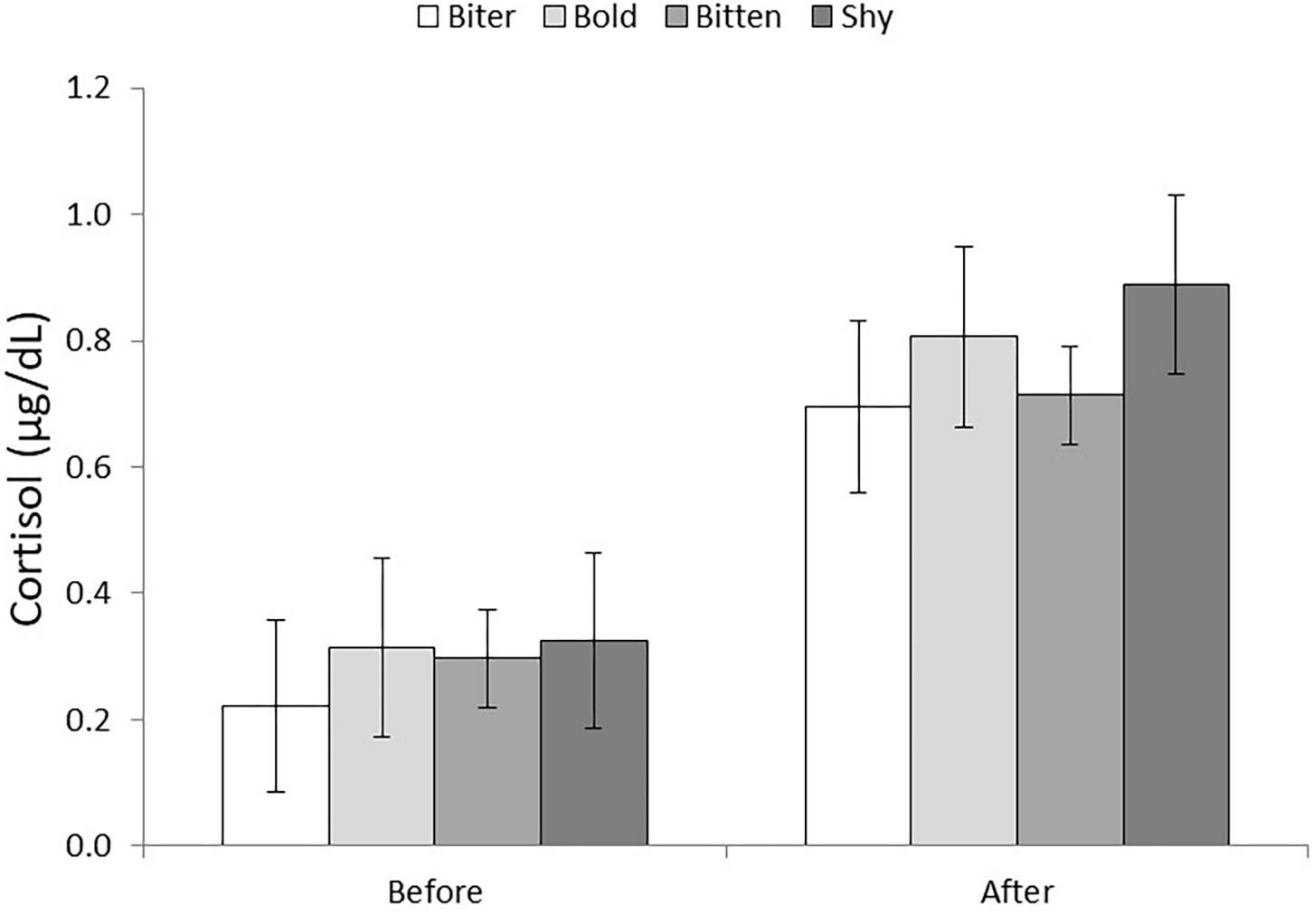
Salivary cortisol levels (μg/dl) of the four pig types (BITER, BOLD, BITTEN, SHY) before and after the open field and novel object tests.

Saliva samples collected after the behavioral tests had higher cortisol levels than prior to the tests (0.289 ± 0.066 vs. 0.776 ± 0.068 μg/dl; *P* < 0.001). There was no effect of pig type on cortisol level, or interaction between pig type and whether the sample was taken before or after the behavior tests (Figure 2). However, the increase in salivary cortisol level was significant for SHY (*P* < 0.05) and BITTEN (*P* < 0.001) pigs, but only tended to increase for BITER and BOLD (*P* = 0.08 for both).

## Discussion

The aim of this work was to investigate firstly whether pigs which were fearful of human contact in their home pen (SHY) would display more fear related behavior in an open field and novel object test than pigs which were not fearful of human contact (BOLD) in the home pen. This was so that we could determine whether the expression of fear related behavior was consistent across scenarios. These data also provided a robust baseline with which to compare our “control” pigs, to bitten and biter pigs, as in previous studies “control” pigs were selected only on the basis that they were neither bitten nor biters. If behavior in the test situation aligned with pig category as determined in the home pen, then we can hypothesize that at least in the fear eliciting situations during which we observed pig behavior (forced human contact and OA and NO tests) these pigs behaved consistently. We then investigated whether pigs that had experienced tail or ear biting, would display the same fear related behaviors as human shy pigs, and whether biter pigs would behave in a similar fashion to human bold pigs. Thus, if the bitten pigs also respond in a similar fashion to SHY pigs in the behavior arena, and the biters in a similar fashion to BOLD, we can hypothesize that these pigs may also be in general more or less fearful across a range of situations. Overall, we found that our hypotheses were partly supported; for some, but not all, measures, pigs which were human shy, and pigs which were bitten, had responses similar to each other, and different to the responses of human bold and biters pigs, and primarily in the OF test. While we are aware of the limitations that our small sample size has created, we believe that this study helps to lay the foundations for the development of further work using a larger sample size, and particularly of biter pigs. Based on our experience with the current study, we also suggest that improvements such as more detailed analysis of vocalizations and the amount of exploration performed in the home pen could add value when selecting pigs for testing. This may help to identify more distinct differences between pig types, and ultimately to determine whether based on their innate personality traits, whether a pig is predisposed to be a biter or a victim.

In agreement with our hypothesis, pigs which we expected would be less fearful in the behavior tests (BOLD and BITER) spent more time exploring the arena, less time by the door, and less time standing still, than other pig types (SHY and BITTEN). Indeed time spent exploring the arena was negatively correlated with both standing still, and time spent by the door. Exploration is considered a normal and highly motivated behavior for pigs, as under natural conditions they spend a high proportion of their time performing exploratory and rooting behavior in their search for food (15). Thus, higher level of performance of this behavior in the human bold, and biter pigs, indicates that their behavior was less inhibited by the potentially fear inducing experience of the open field test than the pigs which were human shy and bitten.

Biter pigs have previously shown to display more exploration behavior in a home pen environment; Ursinus et al. (12) found that time spent exploring pen-mates or the environment was associated with being classified as a tail biter, for finisher pigs which were managed in housing conditions similar to those in our study. Similarly, Zonderland et al. (16) observing that biter pigs interacted with enrichment more than other pen-mates during the 6 days prior to a tail biting outbreak. The pigs in the current study were categorized as being “biters” if they were regularly (>3 times) observed biting over 5 weeks of observation, even though there were no tail biting outbreaks in the pens, and as such they may have simply been fulfilling a high motivation to explore, rather than engaging in compulsive or highly damaging behavior. In contrast, it is possible that bitten pigs may have been less likely to engage in exploration if they were experiencing pain, stress, or sickness behavior or were avoiding biters. Human shy pigs appeared to be consistent in their response to a non-usual situation (a human attempting interaction, and the behavior tests) in that their responses are commonly interpreted as being indicative of fear. In future studies, it would be useful to consider variations in exploration behavior amongst BITER and BITTEN pigs in the home pen prior to the tests.

The lack of the ability of the principle component analysis to reduce the behavior data to meaningful components, as was reported in previous studies (e.g., 4), was disappointing. At the same time, there were yet several correlations between variables, which when examined, can provide some level of insight into the relationship between the measurements. The fact that exploration was negatively correlated with walking, standing still, the number of locations entered and the duration spent at the door is somewhat obvious, as these behaviors are in the main mutually exclusive. Its negative association with the number of screams is also somewhat intuitive, as screams can indicate stressful situations (17). In contrast, grunts are generally considered to be indicative of a “positive” vocalization in pigs.

Nevertheless, in the current study grunts and screams were positively correlated, and both were positively correlated with duration by the door, a behavior which we hypothesize to be related to fear, as well as time spent walking and the number of locations visited in the open field test. These data could indicate that pigs which traveled through the arena without taking time to explore, were attempting to find an escape route. Indeed, Mormede et al. (18) considered that in the novel environment test, fearfulness is usually observed either as low activity (behavioral inhibition) or high activity (behavioral activation, usually associated with escape attempts) together with many squeals. Thus, in this instance, grunting may not represent a positive experience. Marchant et al. (19) found that single grunts could be sub-divided into two types based on sound amplitude profile, with short single grunts being associated with investigatory behavior and on-going single grunts potentially being a form of contact call. Detailed examination of vocalizations was not possible during this study; it may have been more insightful if vocalizations were recorded, and submitted through software which could distinguish the precise components of the sound, as in the studies by Marchant et al. (19) and Schon et al. (20).

Surprisingly, given the associations from the open field test, grunts were associated with a low latency to interact with the novel object, and a longer time interacting with it. Thus, these vocalizations appeared to be more associated with a lack of fear in relation to exposure to a novel object. It is important to remember that the open field and novel object tests may measure different aspects of fear or anxiety. Moreover, grunts and screams were recorded during the former test, and not during the latter. By the time the novel object test occurred, it is possible that the pigs had habituated to the test arena. Moreover, the detailed comparison of fear tests carried out by Andersen et al. (21) identified that aversion to novelty and measures of activity were two separate components. Vocalizations however, were not recorded. Thus, it is possible that vocalizations may or may not have been associated with one or both of these components; in our study, vocalizations were associated with both increased locomotion, and no aversion to novelty.

The tests we used were unfamiliar to all pigs, with no pigs having been previously isolated from their conspecifics. It was therefore unsurprising that cortisol levels were higher after the tests than prior. Although, shy and bitten pigs did not show higher levels than biter or bold pigs either before or after the test, these were the only two categories which had significantly higher levels after the test than before. This confirms our hypothesis that these animals would have a greater stress response to the tests, or indeed the taking of a cortisol sample (which in itself could prove to be fear or stress inducing), than the human bold or biter pigs.

At the time of testing, no major tail biting outbreaks had occurred [e.g., 21.4–25% of pigs per pen with fresh dripping blood or a tail damage score of 3- (22)], and as such the biter pigs may not have been the type of “compulsive” biter that instigates an outbreak, but rather pigs which engage in excessive levels of two stage biting, or sudden forceful biting when aiming to access a resource. Further work, with a larger sample size of “biter” pigs, using pigs which were not tail docked, and using more detailed behavior observations to categorize pig types, would be a worthy exercise.

## Data Availability Statement

The raw data supporting the conclusions of this article will be made available by the authors, without undue reservation.

## Ethics Statement

The animal study was reviewed and approved by Teagasc Animal Ethics Committee (TAEC89/2015). Written informed consent was obtained from the owners for the participation of their animals in this study.

## Author Contributions

AH and KO'D contributed to the conception, design of the study, and completed the writing of the manuscript. AH, J-YC, and KO'D conducted all aspects of the experimental testing. KO'D organized the database and completed all the statistical analysis. All authors contributed to manuscript revision, read, and approved the submitted version.

## Conflict of Interest

The authors declare that the research was conducted in the absence of any commercial or financial relationships that could be construed as a potential conflict of interest.
